# Lessons learnt and lessons missed from flight simulation

**DOI:** 10.1111/eje.12919

**Published:** 2023-07-11

**Authors:** D. J. Allerton, P. Lammertse

**Affiliations:** ^1^ Department of Automatic Control and Systems Engineering University of Sheffield Sheffield UK; ^2^ Retired

**Keywords:** flight simulation, haptic technologies, synthetic training, visualisation, VR in dentistry

## Abstract

This article reviews progress in the development of technologies used in flight simulation and in the training of dentists, drawing out the similarities in training objectives and the limitations of the training devices. It summarises the advances in pilot training with recognised international standards for the construction and acceptance of training devices, noting the impact of flight simulation as a major contributor to the improvements in flight safety. Attention is drawn to the positive transfer of training from synthetic training to airborne operations. The evolution of training methods in dentistry is described covering virtual reality and haptic simulation. The distinction is drawn that tactile feel and visualisation, which is very different from other forms of simulation, is critical to the introduction of synthetic training in dentistry. In particular, progress in methods to provide haptic technologies is reviewed and the importance of novel methods of visualisation, specific to dentistry, are reviewed. This article concludes by outlining progress in flight simulation that is relevant to synthetic training in dentistry but also stresses the differences between the two disciplines. The progress and limitations of flight simulation and the current status and future of synthetic training in dentistry are described, highlighting the potential benefits of lower‐cost haptic devices and the lack of standardisation.

## 1 THE CASE FOR FLIGHT SIMULATION

Flight simulators have been used to train airline pilots since the 1960s.^1^ This development coincided with improvements in the performance of the computers, with many developed by the simulator manufacturers to meet the exacting requirement to simulate aircraft dynamics and produce a realistic view seen from the simulator flight deck. With pilots interacting with the simulator software, all the computing tasks must be completed at least 50 times per second, demanding very high speed computer systems and graphics.

In addition to conversion training to a new aircraft type, the majority of airline pilot training covers recurrent training, which is a compulsory 6‐monthly check of pilot competency to operate the aircraft, takes 2 days, and covers all aspects of aircraft operations. Prior to 1970, much of this training was conducted in airborne exercises, which were potentially life‐threatening if performed incorrectly and, moreover, the cost of training was of the order of 10 times the cost of training in a flight simulator. As the fidelity of these early simulators improved, an increasing number of airlines appreciated the benefits of simulation in terms of both safety and economics to airline operations.^2^ These advances resulted in two major trends. Firstly, as the performance of domestic computers and graphics cards increased, commercial off‐the‐shelf (COTS) systems were used in flight simulation, in contrast to the custom devices produced for the earlier versions of simulators. Secondly, there was a variation in the fidelity (or quality) of the simulators produced by the manufacturers and the training methods used in pilot training.

To resolve the variation between simulation facilities, strict regulations were formulated by the regulators to ensure consistency across the simulators, in particular, to ensure that the simulators accurately modelled the aircraft they simulated, but also to monitor the acceptance tests conducted by an airline in order to confirm that the training provided met rigorous and objective standards. By the 1990s, international regulations^3^ had been adopted and accepted by manufacturers, pilots, airlines, unions and regulators in most countries.

One further development to reduce the cost of flight training was the introduction of part‐task training devices. For example, pilots can train to use a flight management system using a standard laptop computer, rather than a full flight simulator, off‐loading considerable training time on the far more expensive flight simulator. Nowadays, part‐task trainers are used for very specific training tasks, where the emphasis is on the training requirement rather than the realism of the training device. For example, jet‐engine technicians are able to practise on a synthetic computer‐based model of an engine rather than an actual aircraft engine.

## 2 FLIGHT SIMULATION TECHNOLOGIES

With pilots operating a simulator, the real‐time requirement to solve all the equations and provide aircraft displays and a realistic external scene is critical. The implied update rate, known as the frame rate, is typically 50–60 Hz, which is far faster than the human response and ensures that the aircraft motion and visual scene move smoothly, without any discontinuities. Below approximately 20 Hz, a pilot will perceive unnatural jumps and lags in the simulator motion, resulting in a loss of acceptance of the realism of the simulation and significant changes in the pilot response in a simulator.

Nowadays, the realism of the flight deck, including the flight displays, is indistinguishable from the aircraft flight deck and the outside scene is projected so that it is seen through the windscreen of the simulator with natural depth or distance. The content of the external scene is generated by the rendering of complex scenery of terrain and airports using 3D computer graphics and contains sufficient detail to create the visual illusion of flight and taxiing. Even, with modern computers and graphics cards, achieving a 50 Hz frame rate for the visual system is an exacting task. There is a temptation in simulation to trade‐off rendering rate against scene detail but with a fixed frame rate, the only option is to reduce the scene content. However, the fidelity of the external scene must achieve a level of acceptability needed for airline training.

In flight simulation, the motion cues are provided by large hydraulic jacks attached to the base of the flight deck, moving and rotating the complete cabin. Although the motion cues cannot replicate exactly the accelerations in an aircraft, they provide the sensation of motion which is appropriate to transport aircraft. Similarly, actuators provide dynamic loading of the flight controls to give the tactile feel associated with moving the flight controls. Sounds of engines, slipstreams and warnings are generated from sound recordings to provide sounds associated with the full range of flight operations.

## 3 FLIGHT SIMULATION REQUIREMENTS

The regulator has responsibility for approval and qualification of a flight simulator, in effect, answering the question *Does the simulator fly like the aeroplane*? The aircraft and engine dynamics are modelled in detail throughout the flight envelope and this is mostly achieved with modern PC technology and interfaces to the aircraft systems. In some cases, the overall computing performance of the simulator can be increased by using multiple processors, connected via a local network, to meet the frame rate requirement.

The demands on the visual system are particularly challenging in terms of meeting the simulator frame time. The entities in a scene, including terrain, rivers, road, buildings, runways, taxiways and lights are modelled as triangles where the colour and texture of a surface is appended to each triangle, which is rendered in 3D to produce a 2D video image that is displayed by a projector. Considerable effort is required to produce a detailed database of the scene entities and the throughput of graphics operations is only achievable by using modern graphics cards, where each card contains several hundred computing cores.

It is particularly important to provide detail in a scene with accords with ‘real‐life’ events in terms of the position, orientation, colour, texture, shading, resolution and lighting of objects in a scene. By rendering the scene 50 or 60 times per second, the pilot is unaware of any perceptible delay in the visualisation of a scene. Partly as a result of the impetus of games software, it is the combination of real‐time rendering packages and multiple‐core graphics cards that provide real‐time images, to levels of detail that are close to photographic quality.

## 4 TRAINING TRANSFER

In pilot training, it is possible to specify objective tasks and measurable levels of attainment. For example, it may take 25 h of airborne training to fly an instrument approach where the pilot flies solely by reference to the flight instruments, often in conditions of low visibility. To reduce the cost and risk of training in an aircraft, some of this training can be undertaken in a flight simulator. The question then, is – how many hours of simulation would be needed to replace 1 h of airborne training? Note that this transfer of training can be positive, for example, 5 h of training in a simulator can replace 2 h of airborne training, with a potential saving in the cost of training. However, in the case of a poor simulator (or poor training methods) the transfer of training can also be negative, for example, where 5 h of simulator‐based training requires an additional 10 h of airborne training.

However, it is very difficult to predict the transfer of training prior to any simulation. The only objective method to assess the transfer of training is to conduct tests with trainees where one cohort receives only airborne training and their performance is compared with another cohort which receives training that includes simulator‐based training to attain the same level (or standard). This conjecture assumes that specific tasks can be measured objectively. In the previous example of instrument flying, the errors could be measured during an approach, for example, deviations from the desired trajectory. In one study, ab‐initio pilots, using a synthetic training device, reached the solo level in less than 9 h in comparison with pilots trained in an airborne‐only method who took nearly 15 h.^4^ In this example, the training was conducted in the less hostile environment of a flight training device, which was optimised to train specific tasks, relevant to the training requirement.

## 5 EVOLUTION OF SYNTHETIC TRAINING IN DENTISTRY

Dexterity training for dentists before they see patients has a longer history than flight training; for more than a century dental training has taken the form of drilling and restoring extracted or artificial teeth in phantom heads.^5^ Commercial synthetic VR dental training was introduced only as recently as 2010. The reasons for synthetic dental training are very similar to those in flight simulation: the cost and risk of VR training are potentially much lower than those of training in real life, where a threshold level of both graphical and modelling realism was needed before VR training became acceptable.

Synthetic dental training is less mature than flight simulation, and the evidence for transfer and efficacy is still being sought.^6^ Unlike flight training, the reference case in dentistry is not ab initio training on patients, which is clearly infeasible. Instead, VR training is an alternative to the time‐honoured standard of training, which is drilling on plastic teeth.

## 6 REQUIREMENTS OF A DENTAL VR SIMULATOR

Dentistry is an interesting challenge for VR and haptic simulation. The contact forces with the tooth are very slight. Modern restorative dentistry uses composite fillings, and the rotating burr is no longer used as a ‘drill’ to cut square, inversely tapered holes anchoring the amalgam, but primarily ‘brushes away’ decay sparingly, with sideways, stroking motions. The degree of precision required is extreme, with students taking a year or more from the ‘millimetre level’ to reach the ‘0.1 mm level’. Consequently, the haptic system and the visual system need to reflect this accuracy, and they need to be collocated to the same precision. Short‐range depth perception makes stereo imaging almost mandatory, in addition to other depth cues. The sound of the burr is also an important cue to the resistance of the tooth and is relatively easy to simulate. The tone is directly proportional to the RPM of the drill. Two or three sine waves separated by 20 or 30 Hz will be perceived as the typical harsh sound of a dental burr. A simple RPM model for the drill, based on the foot switch position and the simulated burr contact force, are easily modelled on commodity hardware, typically a laptop or a PC.

Haptics has been an active field since around 1985.^7^ The classical haptic device ‘renders’ three degrees of freedom (3‐DOF) XYZ forces to the tip of a swivelling, hand‐held stylus. To the user, these artificially generated forces feel as if they are acting at the tip of a virtual pen. The same pen is shown visually in a virtual world, creating a strong sense of presence in that world; an almost eerie sense of touching and moving objects in a virtual world. Attempts have been made to expand these pen‐based interfaces to render torques as well, but such 6‐DOF devices do not seem to add enough useful functionality to warrant the mechanical complexity.

One would expect the ‘ideal’ generic haptic interface to be a powered glove, giving full‐force feedback to the muscles of the fingers, and full tactile feedback to the skin. Despite numerous attempts, no satisfactory devices have been demonstrated.

Flight simulation points the way to a practical solution. Instead of putting a glove on the user's hand, flight simulators routinely use powered copies of the control sticks and pedals to give a near‐perfect tactile and proprioceptive experience in the cockpit. Using such a handle or ‘manipulandum’ instead of a glove, reduces the number of rendered DOF's to those of the handle rather than those of a glove or indeed the human hand, yielding high‐haptic quality, good tactile experience, and vastly reduced cost. The dental simulation uses the same principle. The haptic device drives a physical copy of the dental handpiece.

Haptic perception is a very different channel from the visual one. At first sight, haptic simulation would seem to be easier than visual simulation. Voluntary hand movements are no faster than 3 Hz, and tool motions are usually limited to three or six degrees of freedom. Modern graphics cards on the other hand render visual images consisting of tens of thousands of individual textured triangles, which are updated at 100 Hz.

However, this view can be misleading. The skin of the human hand is sensitive to a surprising frequency of up to 700 Hz. The human perceptual system uses this information to assess the mass, stiffness and texture of our environment. Touch is used in an active way, where shapes in the environment are explored at a resolution not far below the level of visual fidelity. If the environment is tapped with a finger or a pencil, update rates of at least 1000 Hz are needed to ‘display’ the properly perceived stiffness. Calculating the collisions between two complex shapes is harder (and less well‐developed) than tracing straight visual rays to a single object. The computational requirements of haptics modelling can be high, although they can be mitigated by judicious simplification of the object shapes.

Device design is a non‐trivial problem. Despite improvements in magnetic materials, electric motors have a relatively high mass, and electrical coils have high rise times. Depending on the design, the bandwidth of small robots is closer to 20 Hz or 50 Hz than to 1000 Hz, making it very difficult to simulate the stiffness of a hard stop, or the immediate reaction of a light mass. There is no indication that this limitation will be overcome in the near future, and for extreme requirements, inventive solutions are needed, perhaps using ‘encountered’ devices which do not move the manipulandum directly, but instead move preset stops to locations of expected contact.

## 7 HAPTIC TECHNOLOGIES

There are two basic control paradigms in the design of haptics devices, called *impedance control* and *admittance control*. In many respects the two are duals: the strength of the one is the weakness of the other.^8^ Impedance control is the simpler of the two approaches, but cannot render the stiffness required of a simulated tooth. Admittance control is a slightly more complex technique. It uses a force sensor on the tool tip as an input to a virtual model of the hand‐piece. The tool tip position is not measured in the physical device, as it is in impedance control, but instead, it is calculated in the computer, increasing the precision by orders of magnitude. Admittance control can render any stiffness, and it is the paradigm of choice in dental simulation.

Haptic modelling is an emerging field. There is extensive literature on visual modelling relating directly to dental VR training. The same cannot be said for haptics, and in particular for rendering in admittance control, which is in fact simpler than for impedance control, since all interactions with the virtual environment take place on computed tool positions, which are over a thousand times more accurate than measured device positions.^9^ In all cases, rendering means calculating a contact force between the tool and its simulated environment, and applying this force to the mass of the haptic device. The only difference is that in impedance control, the force is applied by a small motor directly to the physical mass of the device, and in admittance control it is applied to the calculated virtual mass of the tool model.

Contact forces are based on simple spring and friction/damper models. Calculating the intersection depth or volume between a complex tool shape like a burr and a complex environment like a tooth at haptic rates of 1000 Hz can be challenging, but in principle it is a straightforward solution of geometric equations. The spring force is, in effect, a ‘penalty’ on the amount of intersection, hence this method is called ‘penalty based rendering’. In impedance control, an independent visual tool model called a ‘proxy’ is needed to prevent the image of the tool from sinking into the tooth, leading to ‘constraint rendering’, but in admittance control such an extra step is not needed.

Cutting algorithms take the intersection models one step further. Teeth are modelled by cubical voxels of about 0.1 mm grid size. These models are taken from CT scans of actual teeth, augmented with colours based on the local hardness of the material, including pulp, dentin, enamel, and decay. These voxel models are modified in real‐time when the burr is cutting. Improved versions of the Marching Cubes algorithm are used. The teeth are modelled by discrete blocks in the size of CT voxels, which are gradually cut away by the virtual tool.^10^

Updating the shape of the tooth by carving straight sweeps of the burr shape, while at the same time rendering stable haptic force and visual images, is solved by proprietary algorithms.

## 8 HUMAN INTERACTION IN DENTISTRY TRAINING

Visual modelling in medical trainers also differs from flight simulation. Flight simulators display a very large, detailed and complex world, but this is largely a static world, and nearby aircraft are also solid objects. Simulating dental drilling requires active shape change of the teeth in the visual model to a subsample rate of the 1000 Hz haptics model, preferably at the graphics rate of 100 Hz.

Apart from the haptic requirements of the powered DOF's, dental simulation also has some non‐trivial requirements on the passive axes of the tools. The rotation of the hand‐piece around the tip of the burr is unpowered, except in root canal work, which would also require the rendering of torques. Nevertheless, the rotational workspace of the dental hand‐piece is extreme. The hand‐piece is used by left‐handed and right‐handed students, with the burr facing towards all sites of all teeth, and this occurs in both upper and lower jaws. This workspace leaves almost no room for attaching the physical gimbal at the tip of the burr to the haptic device. To make matters worse, there also has to be physical space at all times for the fingers of the student holding the hand‐piece.

The haptic gimbal problem is compounded by the presence of a second stylus in the same workspace, that of the dentist's simulated mirror handle. Besides imaging sites that are not directly visible, dentists frequently use the mirror to push aside the tongue, and they have to avoid tool‐to‐tool collisions between the burr hand‐piece and the mirror handle. Without rendering forces on the mirror handle, so‐called ‘pseudo‐haptics’ may come to the rescue. To a certain extent, visual cues can replace haptic cues by giving users the impression that they are handling soft or lightweight objects. The mirror handle can push the tongue away in the virtual image, without reflecting any real forces to the handle, which is tracked but not powered, but cannot enter any teeth or the burr hand‐piece. For the mirror, this pseudo‐haptics is partially acceptable. The resulting mismatch between the handle and its visual image is resolved unobtrusively once the collision ends.

## 9 VISUALISATION IN DENTISTRY TRAINING

Unlike in haptics, visual displays have improved by many orders of magnitude since the 1980s. Visual resolution down to the sub‐millimetre level is no longer an issue, but collocation is still a major problem. The current generation of VR dental trainers such as the *Simodont* create a collocated image on top of the haptics workspace by the use of a small projection screen viewed via a tilted mirror. Left and right stereo images are projected on the same screen at the proper depth, and separated for the eyes of the student by polarised glasses. It may be expected that the next generation of trainers will use head‐mounted display devices, as soon as they provide the desired accuracy of tracking and acuity of vision.

A fundamental issue in visual rendering is whether to use augmented reality (AR) or full virtual reality (VR). Current generation VR dental trainers have a fully synthetic image, which does not include the student's hands and fingers. This is less unnatural than one might suppose; many students are unaware of this omission, although some dentists find this unacceptable. In real life, the dentist's fingers occlude a sizable part of the view. Leaving out the hands from the image altogether gives the user a better view of the teeth and burr than in real life, which may make the training task ‘too easy’. At the same time, it may reduce the feeling of connectedness to the tool, but this effect tends to go unnoticed, or wears off quickly.

Showing artificially rendered fingers however tends to be worse, falling into a ‘valley of uncanniness’ for most people. Augmented reality with see‐through glasses could solve this problem by showing the actual view of the student's hands including the physical hand‐piece, or at least the stylus part of the handle, surrounded by the synthetic image of the mouth and teeth. The burr head will most likely have to be rendered synthetically, for more accurate collocation between the images of the burr tip and of the tooth under treatment.

Collocation is not simply the same as ‘having everything in one place’. Clearly, if burr contact is felt when the image shows the burr *not* touching the tooth, then any dexterity training effect will be lost or negative; but this is a matter of collocation between the visual *model* and the haptic *model*. Differences of 0.1 mm can be seen and felt. However, this does not mean that humans have a 0.1 mm sense of register between their hands and eyes. If the eyes are closed for a few seconds and the hands are repositioned, jumps in position of several centimetres are acceptable. The human proprioceptive sense, like most other senses, is very sensitive to small changes, but not to absolute values.

Eye surgeons routinely look down through a magnifier loupe or microscope with 5× magnification and a 45° angle for more comfortable head posture. As a rule, parallel translation and scaling between haptics and vision are completely acceptable, and tend to go unnoticed.

Exact collocation of the visual image of the hand‐piece and the actual hand‐piece is only needed insofar as it affects ergonomic posture. In a full VR trainer with no outside vision of the user's hands, static collocation offsets of several centimetres are acceptable, except possibly in depth.

Rotations other than looking up or down however are normally unacceptable, and very difficult to adapt to. Normal users cannot handle a rolling misalignment, and all other rotations need deliberate practice. The effect is similar to using a computer mouse sideways. Experienced dentists are in a league of their own here. They are so used to work with ‘indirect vision’, i.e. by seeing the burr and tooth only via the rotated dental mirror image, that they sometimes do not even notice when the complete artificial visual scene is rolled and rotated by arbitrarily large angles, or indeed mirrored from real life.

There is also a strong temporal component to collocation. The haptics device picks up the user's movements, and the visual image tends to lag by one or two graphics frames. Such unavoidable delays are noticeable and delays exceeding 0.1 s can cause nausea. Even without graphics frame delays, displaying a series of sharp images at graphics rates is unacceptable in all but the slowest of movements, otherwise the moving image becomes a stroboscopic sequence of burr images at successive positions, instead of a single, smoothly moving burr image, ruining any sense of immersion. This problem can only be ameliorated by implementing a motion blur algorithm in the graphics card.

## 10 SIMILARITIES AND DIFFERENCES BETWEEN FLIGHT SIMULATION AND DENTISTRY

Both flight simulation and simulation in dentistry comprise four stages:

- Acquisition of user (inceptor) inputs;
- Computation of the system dynamics;
- Visualisation and projection of the simulation environment; and
- Kinesthetic feedback.

The main requirement with inceptor inputs in a flight simulator is that the data is acquired with sufficient resolution and is used as inputs to the dynamic modelling in the same frame. The system dynamics must be based on a mathematical model^11^ with acceptable fidelity (accuracy) and the underlying equations must be solved at the frame rate.^12^ Similarly, the visualisation software must be computed within the current frame rate, with the added requirement that the details of the scene content and conditions must be appropriate to the simulation application. In flight simulation, the viewpoint translates with the aircraft moving over a mainly static scene with environmental conditions of fog, haze and sunlight. In dentistry, the viewpoint moves with the surgeon's head and eyes, the visualised scene can change dynamically and the environmental conditions include fluid flows, debris from drilling and grinding, water spray and the movement and interaction of surgical tools.

In flight simulation, the dynamics comprises several sets of non‐linear differential equations^13^ whereas finite‐element methods may be used in dentistry simulation to provide the interaction between surgical tools, and physical changes to the tongue, gums and teeth. In flight simulation, the relative cost of training in an aircraft to training in a simulator is so large that it has driven simulator development for nearly 50 years and the use of simulation is generally accepted without question.

A major difference between simulation in aerospace and dentistry is in the methods of projection. A wide‐angle mirror with three or four projectors mounted above the cockpit provides a field‐of‐view very close to an actual aircraft flight deck, without the need for stereo projection. For some military aircraft, helmet‐mounted displays are used. While providing full 360° vision in all directions, problems can arise with tracking both the head position and also the retina of the pilot's eyes, particularly from delays in acquiring position inputs, but also from slippage of the helmet and changes in the eye geometry under conditions of stress. Static wide‐angle projection is not feasible in dentistry applications and emphasis is given to stereo head‐ and eye‐mounted displays. The cost of high fidelity displays and accurate tracking is considerable and, in some case, a compromise is made to reduce the cost of tracking systems, to the detriment of the effectiveness of the simulation.

In addition, the haptic feel associated with flight controls can be implemented with relatively large motors or hydraulics in flight simulation. However, the power and bandwidth available for hand‐held haptics is relatively small, making it difficult to replicate the range and resolution of applied forces.

## 11 ADVANCES IN FLIGHT SIMULATION

Arguably, the major advance in flight simulation has been the standardisation arising from the worldwide acceptance of international regulations. Simulator manufacturers understand the levels of fidelity required for training devices, aircraft manufacturers are able to acquire (at considerable cost to an airline) very accurate flight data to provide the basis of high fidelity airframe and engine dynamics and processes are well defined for regulators and operators to validate simulation facilities and for their operation in an airline.

In the last 10–20 years, the cost of computer systems, electrical actuators and computer graphics has reduced, while at the same time, the performance and reliability of COTS items needed in simulation has increased enormously. For the simulator manufacturers, the development of a simulator is largely the assembly of components from bought‐in COTS items and customisation of software to achieve acceptable fidelity.

**FIGURE 1:**
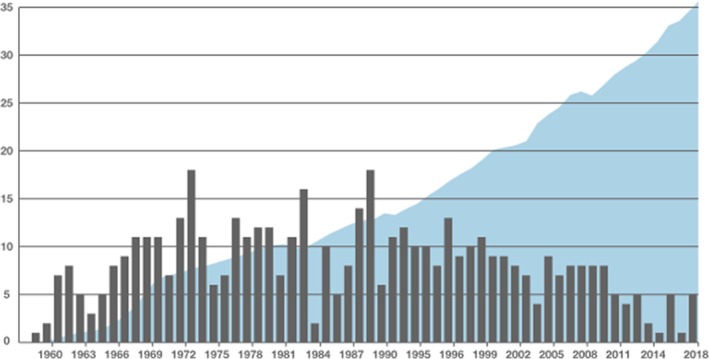
Worldwide aircraft accidents since 1960. The bars show the number of fatal aircraft incidents and the shaded region shows the increase in number of flights per year.

It can also be claimed that flight simulation has made a major contribution to flight safety. Figure 1 shows the fatal accidents for commercial aircraft since 1960,^14^ where the grey bars show the annual number of fatal aircraft accidents worldwide and the shaded background region shows the increase in the number of flights per year.

Since the widespread adoption of flight simulation in the 1970s, the number of flights has increased by a factor of four, whereas the number of annual accidents has reduced by a factor of approximately two. Although no studies have been undertaken on the impact of flight simulation and international standards, the increased quality of training afforded by simulation has undoubtedly reduced the accident rate. In compulsory 6‐monthly currency checks, pilots practice for the full range of emergencies and operational conditions that can occur in airline operations. Lessons learnt from air accident investigations are fed back to training organisations and incorporated into training programmes. Such has been the success of flight simulation, that accredited airlines are able to provide zero flight‐time (ZFT) training, where all the conversion training is undertaken in the simulator. The trainee's first flight in the aircraft is with fee‐paying passengers, albeit under the guidance of a training Captain.

## 12 LIMITATIONS OF FLIGHT SIMULATION

Although the cost of flight simulators has dropped, a typical simulator costs in excess of $10 million and consequently, flight simulators are restricted to airlines and military organisations. However, the majority of pilots worldwide are private and commercial pilots operating aircraft with a mass less than 12 500 lb, with no access to simulator‐based training. There are examples of lower‐cost simulators developed for flight training schools, but these organisations face two problems. Firstly, time spent training in lower fidelity simulators is not recognised as valid hours towards a licence, so there is no incentive to reduce the training in an aircraft. Secondly, the cost of procurement of a simulator is sufficiently high that a flying school is unlikely to recover its investment costs.^15^ By comparison, approved airline simulators can generate significant income for an airline by leasing spare training slots to other airlines or training organisations.

Flight simulation up to the 1970s was technology‐driven, with a solid scientific base. However, since that era, it can be argued that flight simulation has become a major business in the aerospace industry with simulators assembled from COTS items, with little emphasis or encouragement for research. In particular, there has been minimal research on the effectiveness of flight simulation in flight training.^16^ This situation is understandable; most airline simulators are operated 23 h per day, throughout the year and few research organisations or universities can afford a research facility with a simulator costing $10 million. Consequently, studies into the effectiveness of simulation are sparse and often limited to prototype training devices.^17^ With this lack of evidence, it is hardly surprising that regulators insist on the highest level of fidelity, based on the assumption that realism can ensure training effectiveness. Whereas, some studies, particularly with part‐task trainers, tend to contradict this view. Currently, there is no answer to the question, *Can lower fidelity devices provide effective training at much lower training costs*?

## 13 THE STATUS AND FUTURE OF SYNTHETIC TRAINING IN DENTISTRY

The first generation of haptic dental trainers is now in service, and it is time to review the original requirements. Replacing (part of) the phantom heads by VR trainers had the objective of making dental training more effective, and in particular, to make it more *cost* effective. Experienced teachers for dental training are hard to come by, if only because salaries are significantly higher in private practice than in academia. But even where teachers are available, the feedback to students is mostly based on a finished result – the ‘restored’ plastic tooth. Haptic training devices on the other hand can be programmed to monitor the actual behaviour of the student during drilling, giving far more timely feedback. Also, a far greater variety of cases can be presented than in the limited library of plastic teeth. There is a potential for a better learning process, but this is not fully realised by the current generation of training devices. Also, a move from preclinical to clinical synthetic training is currently happening as intra‐oral scans of the patient can be uploaded into the simulation environment. This enables training in advance on a virtual copy of the specific clinical situation optimising the preparation of the student for the specific task and avoiding unnecessary clinical accidents resulting in increased clinical safety.^18^

Students do not perceive haptic VR training to be as ‘real’ as plastic teeth. Actual burrs, with actual water spray, are closer to what students are expecting to learn to use, even though to a real dentist the hardness of the plastic teeth, and especially the variation between dentin and enamel, is less realistic than the VR experience.

There are two possible future directions. One is towards even higher quality and increased use of automated grading and monitoring during practice. This is attractive but is unlikely to drive down device costs. The other direction is lowering cost, perhaps at the expense of some of the haptic quality. Sacrificing off‐design conditions, such as high forces and brisk motions, may allow low‐cost devices, which still have satisfactory training effectiveness under nominal conditions, to be introduced. This approach would also open up the possibility of home use for students.

Haptic interfaces, even the simple ones, currently tend to be far more expensive than visual interfaces, by a factor of 10. Admittance‐controlled devices are at the high end of this range. This situation limits the use and development of haptics by hobbyists, creating a cycle where cost remains high and rendering software is less readily available than visual software. There is no technical reason why this cost cycle cannot be broken, but a new impetus is needed, which could be in the form of a new commercial device in the cost range of a few hundred dollars, for example, the once promising Novint Falcon. New paradigms including pseudo‐haptics or encountered devices could be explored more easily when haptic rendering algorithms are widely available and easily tested on commodity haptic hardware. Low‐cost devices can help break the cost cycle holding back the development of haptics if this threshold can be crossed.

## 14 CONCLUSIONS

Flight simulation has been used in training and checking of airline pilots for over 30 years and, arguably, has made a fundamental improvement to the safety of airline operations. These training devices are accepted by manufacturers, operators, regulators, flight crew and pilot unions. As a consequence of improvements in the provision of motion and visual cues, regulations have come into force covering the design, implementation and training in airlines, which are accepted worldwide. The coming years will continue to see improvements in visual fidelity and possibly the introduction of lower cost fixed‐based training devices to increase the participation of commercial pilots operating light aircraft.

The situation in training in dentistry is different. The use of synthetic training is in an early phase and there is no standardisation of training devices or training methodologies. There are also major questions over the most appropriate forms of visualisation and haptic devices, in particular, where the required fidelity exceeds the performance of current devices. It is not clear how training in dentistry will develop, particularly where conventional training methods can provide an acceptable solution. Arguably, there is a case to conduct trials to validate the effectiveness of current training devices and, with advances in head displays and stereo visualisation and improvement in motor drive technologies, it is possible that training in dentistry will see the benefits of these new technologies.

## CONFLICT OF INTEREST STATEMENT

None declared.

## Data Availability

The data supporting the findings of this study are available from the authors upon request.
